# Associations of elevated glucose levels at each time point during OGTT with fetal congenital heart diseases: a cohort study of 72,236 births

**DOI:** 10.1186/s12884-023-06152-w

**Published:** 2023-12-05

**Authors:** Qian Zhang, Shuhua Lai, Yulong Zhang, Xu Ye, Yi Wu, Tinghua Lin, Huiyun Huang, Wenhui Zhang, Hai Lin, Jianying Yan

**Affiliations:** 1 Department of Epidemiology and Health Statistics, School of Public Health, Fujian Medical University, Fuzhou, 350004 Fujian China; 2 Department of Neonatology, Fujian Maternity and Child Health Hospital College of Clinical Medicine for Obstetrics & Gynecology and Pediatrics, Fujian Medical University, Fuzhou, 350001 Fujian China; 3 Department of Obstetrics and Gynecology, Fujian Maternity and Child Health Hospital College of Clinical Medicine for Obstetrics & Gynecology and Pediatrics, Fujian Medical University, Fuzhou, 350001 Fujian China

**Keywords:** Glucose, Gestational diabetes mellitus, OGTT, Congenital heart disease

## Abstract

**Background:**

It remains unclear how the condition of glucose metabolism during pregnancy affects fetal outcomes. This study aimed to investigate the associations of gestational diabetes mellitus (GDM) and elevated glucose levels at each time point during oral glucose tolerance test (OGTT) with congenital heart disease (CHD) risk in offspring.

**Methods:**

We conducted a retrospective cohort study of mothers with singleton pregnancies of 20 weeks or more registered at Maternal and Child Health Centers in Fujian Province, China. The OGTT results and offspring CHD occurrence were collected. We used logistic regression to analyse the association between elevated blood glucose at each time point during OGTT and CHD.

**Results:**

A total of 71,703 normal and 533 CHD fetuses were included. Compared to the corresponding normal group, women with GDM, elevated blood glucose at different time points in OGTT (0 h ≥ 5.1 mmol/L, 1 h ≥ 10 mmol/L, and 2 h ≥ 8.5 mmol/L) showed an increased risk of CHD in offspring (adjusted OR = 1.41, 1.36, 1.37, and 1.41, all *P* < 0.05, respectively). Compared to group 1 (normal OGTT 0 h, 1 h and 2 h), the risk of CHD was higher in group 3 (normal OGTT 0 h and abnormal OGTT 1 h or 2 h) and group 4 (abnormal OGTT 0 h, 1 h and 2 h), OR = 1.53 and 2.21, all *P* < 0.05, respectively. Moreover, we divided participants by advanced maternal age, multipara, assisted reproduction, fetal sex, and others, similar associations were observed in the subgroup analyses.

**Conclusion:**

Elevated blood glucose at different time points during OGTT was associated with CHD in offspring. Fetuses of pregnant women with GDM should be screened for a high risk of CHD.

## Introduction

Diabetes mellitus is a metabolic disorder that is increasingly prevalent among women of reproductive age [1]. Maternal diabetes can be categorized into two subtypes: pre-gestational diabetes mellitus (PGDM) and gestational diabetes mellitus (GDM). GDM is characterized by any degree of glucose intolerance that occurs or is first detected during pregnancy [2]. It is one of the most common complications of pregnancy, affecting 9–26% of the obstetric population [3, 4]. A meta-analysis conducted in 2018 showed that the combined prevalence of GDM in the Asian population was approximately 11.5% (95%CI: 10.9%-12.1%), and this number is expected to continue increasing in the future [5]. This suggests that a growing number of pregnant women will be affected by GDM.

GDM is emerging as an important public health concern due to its association with severe perinatal outcomes, such as perinatal death and congenital malformations [6–8]. Maternal diabetes-induced congenital malformations can affect any developing organ or system. Congenital heart disease (CHD) is one of the most common congenital diseases, affecting the health of millions of newborns annually [9]. The pathogenesis of CHD is complex, and its exact etiology is largely unknown. PGDM has been identified as a potential risk factor for CHD, while the relationship between GDM and CHD risk remains to be fully established. Data from multiple prospective cohort studies indicate that mothers with GDM are more likely to have offspring with CHD than those without GDM [10–12]. Hyperglycemia can affect the development of the fetal heart, leading to structural and functional abnormalities [13, 14]. Other studies have found that even women with lesser degrees of hyperglycemia have a significantly higher risk of poor pregnancy outcomes than those without diabetes [15]. Additionally, among non-diabetic women, diets with a proportionally high glycemic index or high glycemic load increase the risk of birth defects [16, 17]. These findings indicate that blood glucose levels can have an important impact on the development of embryos. Unfortunately, elevated blood glucose is a persistent risk factor, and there is no clear-cut-off value that separates low-risk groups from high-risk groups. Particularly, the associations of basal blood glucose and postprandial blood glucose with fetal CHD remain unclear. Considering that basal blood glucose and postprandial blood glucose levels reflect different mechanisms of glucose metabolism disorders, further investigation is required.

Thus, we conducted a retrospective hospital-based cohort study to investigate the association between GDM diagnosed at different time points in oral glucose tolerance test (OGTT) and the risk of fetal congenital heart diseases in pregnancy.

## Materials and methods

### Study population and data collection

This is a retrospective cohort study conducted in the Fujian Provincial Maternity and Children’s Hospital, China, from January 2014 to December 2020, using data obtained from national neonatal networks. The study population included singleton pregnant women with pregnancies at or above 24 weeks gestation. Maternal age, hypertensive disorders during pregnancy, glucose tolerance test results, delivery mode, neonatal characteristics, and birth outcomes were among the information collected. The exclusion criteria were cases that lacked significant medical history, including ambiguity of conceptual age, maternal diseases such as gestation weeks < 20 or > 44, pre-gestational diabetes, fever (> 38 °C), pesticide exposure within 3–8 weeks of gestation, history of inbreeding, or genetic defects, and multiple pregnancies. Participants were informed of the purpose of data collection, and the collection and transmission of data were approved by the Ethics Committee of Fujian Women and Children's Hospital (IRB NO. 2020–2049), all methods were performed in accordance with the relevant guidelines and regulations.

### Main exposure

OGTT were performed at 24 to 28 weeks of gestation and at the first visit after 28 weeks. The 75 g OGTT method: fasting for at least 8 h and eating normally for 3 consecutive days, that is, eating no less than 150 g of carbohydrates per day. No smoking during the inspection. During the trial, 75 g glucose solution 300 ml was taken orally within 5 min. The venous blood of pregnant women was taken three times before oral glucose, one hour and two hours after glucose (calculated from drinking glucose water), and put into the test tube containing sodium fluoride. The glucose oxidase method was used to measure blood glucose levels. Diagnostic criteria: Blood glucose levels should be below 5.1 mmol/L, 10.0 mmol/L and 8.5 mmol/L (92 mg/dL, 180 mg/dL and 153 mg/dL) before and 1 and 2 h after glucose drinking intake, respectively. Any blood glucose level that meets or exceeds these criteria is diagnosed as gestational diabetes.

### Outcome definition

Fetal malformations were confirmed by echocardiography, cardiac catheterization, or above combined with ultrasound and reference data, cytogenetic examination, drug administration records, and follow-up data. The outcomes of this study were CHD in the offspring, including six subtypes: patent ductus arteriosus, ventricular septal defect, atrial septal defect, pulmonary artery related malformation, aortic associated malformation, and other cardiac malformations. The concomitance of cardiovascular abnormalities was recognized and counted only if these abnormalities were embryologically independent from each other. Temporarily isolated cardiovascular conditions in neonates, such as patent oval foramen, were considered to be normal, and neonates with identified trisomies, heterotaxy syndrome, or abnormal cardiac looping were not counted.

### Statistical analysis

Continuous variables were described by mean ± standard deviation or median (range quartiles), and comparisons between groups were made by using the t-test or one-way analysis of variance (ANOVA) or Mann–Whitney U test or Kruskal–Wallis rank sum test. Categorical variables were described by frequencies (percentages) and comparisons between groups were made using the chi-square test. The Bonferroni correction was used in multiple tests. CHDs were divided into two subgroups according to the classification of congenital malformations: the atrial or ventricular septal defect (AVSD) group and the other group for statistical analysis. In order to reflect the glucose metabolism patterns in pregnant women, the subjects were divided into four groups according to their blood glucose at OGTT 0 h, 1 h and 2 h: Group 1 indicates that OGTT 0 h, 1 h and 2 h are normal at the same time. Group 2 indicates that OGTT 0 h is abnormal, while OGTT 1 h and 2 h are normal. Group 3 indicates that OGTT 0 h is normal, while OGTT 1 h or 2 h is abnormal. Group 4 indicates that OGTT 0 h, 1 h and 2 h are all abnormal. Tests for trend were conducted with regressions by entering the OGTT cross-group variable as a continuous variable in the models. Binary or multinomial logistic regression was used to assess the relationship between GDM, abnormal blood glucose at different time points in OGTT and the risk of CHD and its subtypes. Subgroup analyses were conducted for factors such as elderly parturient, multipara, assisted reproduction, gestational hypertension, premature birth, and sex of newborn infant. We also tested potential interactions between subgroups using the likelihood ratio test. All analyses were performed using R version 4.1.0, and statistical significance was established when *P* < 0.05.

## Results

### Basic characteristics

**Fig. 1:**
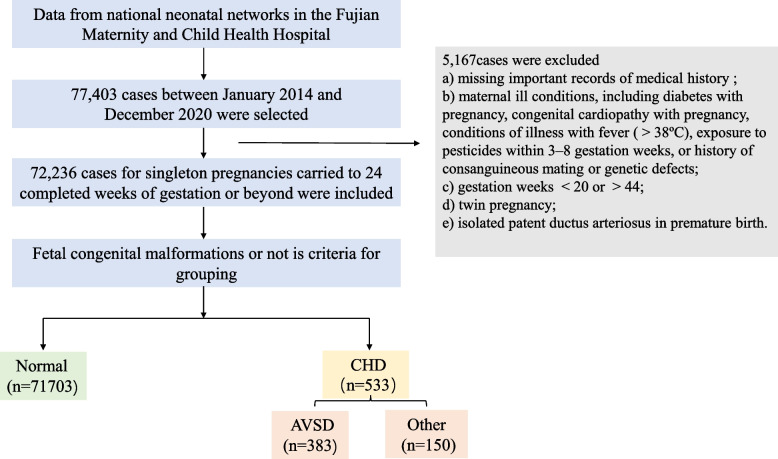
Flow chart of study total results to the inclusion or exclusion

In the study, a total of 77,403 pregnant women were followed up by the Fujian Provincial Maternal and Child Health Hospital from 1 January 2014 to 31 December 2020. After excluding 5,167 pregnant women who did not meet the inclusion criteria, the final cohort included 72,236 women. Among them, 71,703 whose offspring did not have congenital malformations were classified in the normal group, while 533 whose offspring had CHD were classified in the CHD group. The CHD group was further divided into two subgroups based on the classification of congenital malformations: the AVSD group (383) and the other group (150). The flow chart of the subjects included is shown in Fig. 1.

### Clinical characteristics

On average, the pregnant women in the study were 30.2 ± 4.3 years old. Of the pregnant women, 16.2% were elderly parturients, and 44.0% were multiparous women. The average gestational week of delivery was 38.9 ± 1.5 weeks, and the rates of gestational hypertension and premature birth were 3.3% and 4.8%, respectively. Pregnant women in the normal group were younger and had a lower incidence of gestational hypertension and premature birth compared to those in the CHD group (*P* < 0.05) (Table 1).

**Table 1:** Characteristics of the studied sample of mothers and their offspring

Variables	Total (*n* = 72236)	Normal (*n* = 71703)	CHD			*P value*^*#*^
			**All CHD** **(*n* = 533)**	**AVSD** **(*n* = 383)**	**Other** **(*n* = 150)**	
Maternal age, year	30.2 ± 4.3	30.2 ± 4.3	30.6 ± 4.5	30.9 ± 4.5	29.9 ± 4.5	0.036
Elderly parturient, n (%)						0.03
No	60,558 (83.8)	60,130 (83.9)	428 (80.3)	303 (79.1)	125 (83.3)	
Yes	11,678 (16.2)	11,573 (16.1)	105 (19.7)	80 (20.9)	25 (16.7)	
Multipara, n (%)						0.024
No	40,488 (56.0)	40,163 (56)	325 (61)	224 (58.5)	101 (67.3)	
Yes	31,748 (44.0)	31,540 (44)	208 (39)	159 (41.5)	49 (32.7)	
Gravidity, n (%)						0.674
1	29,518 (40.9)	29,307 (40.9)	211 (39.6)	149 (38.9)	62 (41.3)	
2	22,257 (30.8)	22,095 (30.8)	162 (30.4)	115 (30)	47 (31.3)	
≥ 3	20,461 (28.3)	20,301 (28.3)	160 (30)	119 (31.1)	41 (27.3)	
Assisted reproduction, n (%)						0.22
No	70,457 (97.5)	69,942 (97.5)	515 (96.6)	370 (96.6)	145 (96.7)	
Yes	1779 ( 2.5)	1761 (2.5)	18 (3.4)	13 (3.4)	5 (3.3)	
Gestational hypertension, (%)						0.034
No	69,832 (96.7)	69,326 (96.7)	506 (94.9)	360 (94)	146 (97.3)	
Yes	2404 ( 3.3)	2377 (3.3)	27 (5.1)	23 (6)	4 (2.7)	
Gestational week of delivery, week	38.9 ± 1.5	38.9 ± 1.4	38.4 ± 2.2	38.3 ± 2.2	38.7 ± 2.0	< 0.001
Premature birth, n (%)						< 0.001
No	68,784 (95.2)	68,334 (95.3)	450 (84.4)	318 (83)	132 (88)	
Yes	3452 ( 4.8)	3369 (4.7)	83 (15.6)	65 (17)	18 (12)	
Sex of newborn infant, n (%)						0.297
Boys	38,688 (53.6)	38,415 (53.6)	273 (51.2)	199 (52)	74 (49.3)	
Girls	33,548 (46.4)	33,288 (46.4)	260 (48.8)	184 (48)	76 (50.7)	

Data are presented as mean ± standard deviation, or median (range interquartile), or as number (percentage)

*Abbreviations*: *AVSD* atrial or ventricular septal defect, *CHD* congenital heart defect

^#^: *P* value at the last column indicates the comparison of normal (column 3) and all CHD group (column 4) by using the independent samples t-test or Mann–Whitney U test

**Fig. 2:**
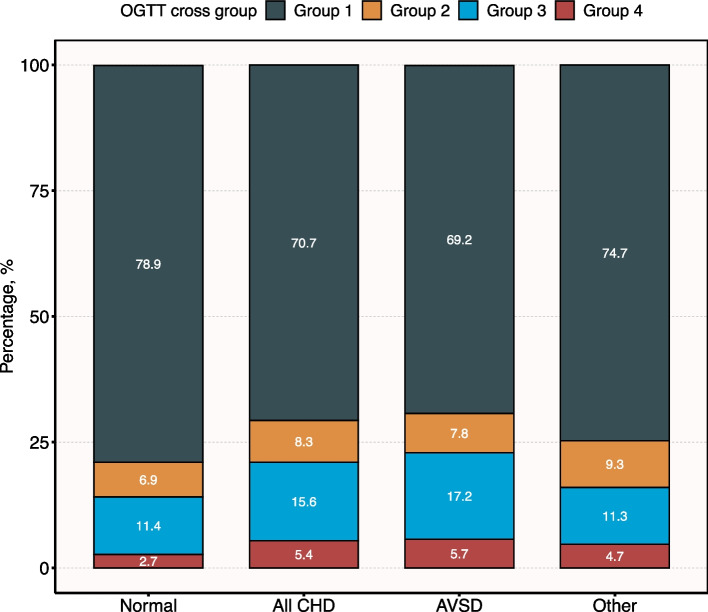
Plot of mid-pregnancy glucose status according to congenital heart defect.  OGTT cross group: Group 1 represents OGTT 0 h, 1 h and 2 h are all normal; Group 2 indicates that OGTT 0 h is abnormal, while OGTT 1 h and 2 h is normal; Group 3 indicates that OGTT 0 h is normal, while OGTT 1 h or 2 h is abnormal; Group 4 represents OGTT 0 h, 1 h or 2 h are all abnormal

Figure 2 displays the plot of mid-pregnancy glucose status according to CHD in offspring. The proportion of normoglycaemic in the CHD group was lower than in the non-CHD group (70.7% vs. 78.9%, *P* < 0.05). The main feature of maternal glucose abnormalities in offspring with CHD is a normal OGTT 0 h and an abnormal OGTT 1 h or 2 h, which accounted for 15.6% in the all CHD group, 17.2% in the AVSD group, and 11.3% in the other group.

### Associations of mid-pregnancy glucose status with CHD in offspring

Table 2 shows the results of the binary logistic regression analysis, examining the relationships between mid-pregnancy glucose status and the risk of CHD in offspring. In the crude models, compared to the corresponding normal reference, women with GDM, elevated glucose at different time points in OGTT (0 h ≥ 5.1 mmol/L, 1 h ≥ 10 mmol/L, and 2 h ≥ 8.5 mmol/L) showed an increased risk of CHD in offspring (OR = 1.55, 1.48, 1.54, and 1.59, all *P* < 0.05, respectively). After adjusting for maternal age, gestational hypertension, gravidity, parity, gestational week of delivery, and sex of the newborn infant, a statistically significant relationship between these factors and CHD risk in offspring still existed (*P* < 0.05).

**Table 2:** Binary logistic regression analysis of the relationships between mid-pregnancy glucose status and congenital heart defect

**Variable**	**n(%)**	**Crude**		**Adjusted**^a^	
		***OR (95%CI)***	***P value***	***OR (95%CI)***	***P value***
GDM					
No	377 (0.7)	1 (Ref)		1 (Ref)	
Yes	156 (1.0)	1.55 (1.28 ~ 1.87)	** < 0.001**	1.41 (1.17 ~ 1.71)	** < 0.001**
OGTT 0 h					
< 5.1 mmol/L	460 (0.7)	1 (Ref)		1 (Ref)	
≥ 5.1 mmol/L	73 (1.0)	1.48 (1.15 ~ 1.9)	**0.002**	1.36 (1.06 ~ 1.75)	**0.016**
OGTT 1 h					
< 10 mmol/L	456 (0.7)	1 (Ref)		1 (Ref)	
≥ 10 mmol/L	77 (1.1)	1.54 (1.21 ~ 1.96)	**0.001**	1.37 (1.07 ~ 1.75)	**0.013**
OGTT 2 h					
< 8.5 mmol/L	460 (0.7)	1 (Ref)		1 (Ref)	
≥ 8.5 mmol/L	73 (1.1)	1.59 (1.24 ~ 2.04)	** < 0.001**	1.41 (1.09 ~ 1.82)	**0.008**
OGTT status^b^					
Group 1	377 (0.7)	1 (Ref)		1 (Ref)	
Group 2	44 (0.9)	1.33 (0.97 ~ 1.82)	0.077	1.26 (0.92 ~ 1.72)	0.152
Group 3	83 (1)	1.52 (1.2 ~ 1.93)	**0.001**	1.39 (1.09 ~ 1.78)	**0.007**
Group 4	29 (1.5)	2.21 (1.51 ~ 3.23)	** < 0.001**	1.86 (1.26 ~ 2.74)	**0.002**
*P for trend*			** < 0.001**		** < 0.001**

*Abbreviations*: *CHD* congenital heart defect, *GDM* gestational diabetes mellitus, *OGTT* oral glucose tolerance test

^a^ Adjusted for infant sex, maternal age, assisted reproduction, gestational hypertension, gravidity, parity, and gestational week of delivery

^b^ OGTT status: Group 1 represents OGTT 0 h, 1 h and 2 h are all normal; Group 2 indicates that OGTT 0 h is abnormal, while OGTT 1 h and 2 h is normal; Group 3 indicates that OGTT 0 h is normal, while OGTT 1 h or 2 h is abnormal; Group 4 represents OGTT 0 h, 1 h or 2 h are all abnormal

The study also analyzed the differences in the incidence of CHD in offspring under different combinations of OGTT 0 h, 1 h, and 2 h test results. In the adjusted model, Group 3 (OGTT 0 h normal and OGTT 1 h or 2 h abnormal) and Group 4 (OGTT 0 h, 1 h, and 2 h all abnormal) showed a higher risk of CHD in offspring compared to Group 1 (OGTT 0 h, 1 h, and 2 h all normal) (OR = 1.39, 95%CI: 1.09–1.78; OR = 1.86, 95%CI: 1.26–2.74, respectively). However, Group 2 (OGTT 0 h abnormal, OGTT 1 h and 2 h normal) did not show a statistically significant increase in CHD risk relative to Group 1. Furthermore, the risk of CHD in offspring tended to increase as the group increased from 1 to 4.

### The associations between mid-pregnancy glucose status and CHD subtypes in offspring

Multinomial logistic regression analysis was performed to explore the associations between mid-pregnancy glucose status and CHD subtypes in offspring, as shown in Table 3. After adjusting for confounding factors, GDM and abnormal OGTT 1 h were significantly positively associated with AVSD risk (OR = 1.48, 95%CI: 1.18–1.84; OR = 1.6, 95%CI: 1.22–2.11, respectively). However, the effect size of abnormal OGTT 0 h and 2 h was relatively smaller and only marginally significant. Furthermore, a significant positive correlation between abnormal OGTT 2 h and other CHD subtype risk was also observed (OR = 1.63, 95%CI: 1.02–2.62). The results of cross-grouping OGTT 0 h, 1 h, and 2 h with the risk of offspring AVSD were consistent with total CHD, with the risk of offspring AVSD increasing with increasing groups from 1 to 4.

**Table 3:** Multinomial logistic regression analysis of the associations between mid-pregnancy glucose status and specific congenital heart defect subtypes

**Variable**	**AVSD**			**Other**^a^		
	***n (%)***	***OR (95%CI)***	***P value***	***n (%)***	***OR (95%CI)***	***P value***
GDM						
No	265 (0.5)	1 (Ref)		112 (0.2)	1 (Ref)	
Yes	118 (0.8)	1.48 (1.18 ~ 1.84)	**0.001**	38 (0.2)	1.25 (0.86 ~ 1.82)	0.242
OGTT 0 h						
< 5.1 mmol/L	331 (0.5)	1 (Ref)		129 (0.2)	1 (Ref)	
≥ 5.1 mmol/L	52 (0.7)	1.32 (0.98 ~ 1.77)	0.069	21 (0.3)	1.47 (0.93 ~ 2.35)	0.102
OGTT 1 h						
< 10 mmol/L	318 (0.5)	1 (Ref)		138 (0.2)	1 (Ref)	
≥ 10 mmol/L	65 (0.9)	1.6 (1.22 ~ 2.11)	**0.001**	12 (0.2)	0.77 (0.42 ~ 1.4)	0.388
OGTT 2 h						
< 8.5 mmol/L	331 (0.5)	1 (Ref)		129 (0.2)	1 (Ref)	
≥ 8.5 mmol/L	52 (0.8)	1.33 (0.99 ~ 1.8)	0.061	21 (0.3)	1.63 (1.02 ~ 2.62)	**0.041**
OGTT status^b^						
Group 1	265 (0.5)	1 (Ref)		112 (0.2)	1 (Ref)	
Group 2	30 (0.6)	1.21 (0.83 ~ 1.77)	0.333	14 (0.3)	1.39 (0.8 ~ 2.43)	0.247
Group 3	66 (0.8)	1.53 (1.16 ~ 2.01)	**0.003**	17 (0.2)	1.05 (0.63 ~ 1.76)	0.858
Group 4	22 (1.1)	1.91 (1.23 ~ 2.99)	**0.004**	7 (0.4)	1.72 (0.79 ~ 3.74)	0.169
*P for trend*			** < 0.001**			0.257

All models adjusted for infant sex, maternal age, assisted reproduction, gestational hypertension, gravidity, parity, and gestational week of delivery

*Abbreviations*: *AVSD* atrial or ventricular septal defect, *GDM* gestational diabetes mellitus, *OGTT* oral glucose tolerance test

^a^ Congenital heart defect other than atrial or ventricular septal defect, and patent ductus arteriosus

^b^ OGTT status: Group 1 represents OGTT 0 h, 1 h and 2 h are both normal; Group 2 indicates that OGTT 0 h is abnormal, while OGTT 1 h and 2 h is normal; Group 3 indicates that OGTT 0 h is normal, while OGTT 1 h or 2 h is abnormal; Group 4 represents OGTT 0 h,1 h and 2 h are all abnormal. Adjusted for infant sex, maternal age, assisted reproduction, gestational hypertension, gravidity, parity, and gestational week of delivery

### Subgroup analysis

**Fig. 3:**
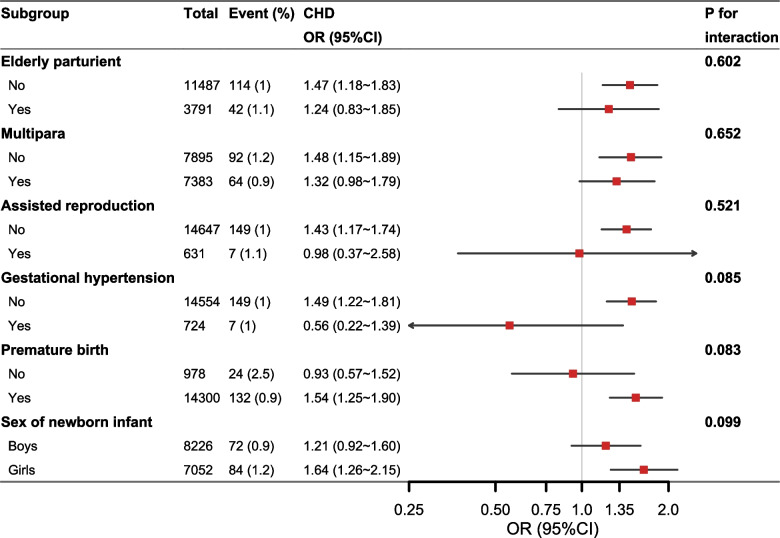
Subgroup analysis of the associations between gestational diabetes mellitus and risks of congenital heart defect

**Fig. 4:**
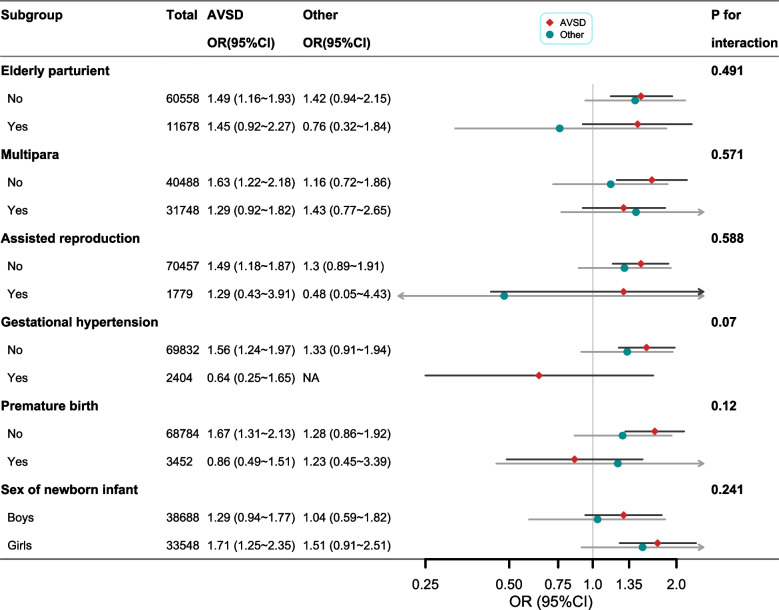
Subgroup analysis of the associations between gestational diabetes mellitus and risks of congenital heart defect subtypes. Multinomial logistic regressions adjusted for infant sex, maternal age, assisted reproduction, gestation hypertension, gravidity, parity, and gestational week of delivery (not for adjusted variables stratified analysis)

The results of the subgroup analysis showed that the association between GDM and CHD risk in offspring was statistically significant in non-elderly women, non-multiparous women, non-assisted reproduction women, non-gestational hypertension women, preterm birth women, and pregnant women with female newborns (Fig. 3). Although there were differences in significance among the partial subgroups, none of the interaction effects were statistically significant (all P > 0.05). Furthermore, the results for disease subtypes revealed that this statistical association was primarily associated with AVSD risk and was not associated with other subtypes (Fig. 4).

## Discussion

This retrospective cohort study investigated whether elevated blood glucose at different time points in OGTT during mid-pregnancy was associated with an increased risk of CHD in offspring, particularly AVSD. In the current study, we found that maternal metabolic disorders of blood glucose were indeed associated with an increased risk of fetal CHD. Moreover, it was also found that elevated fasting glucose alone did not significantly increase the risk of CHD in the offspring, whereas pregnant women with both fasting and post-prandial glucose abnormalities demonstrated a statistically significant higher risk.

The association between maternal diabetes mellitus and congenital defects in offspring has long been a topic of interest. A recent meta-analysis [18] synthesized 52 studies from multiple geographic regions exploring the impact of maternal diabetes on offspring CHD and found that the study region was the main source of heterogeneity in the association between the two. So far, most epidemiological studies have focused on North America, while there are fewer relevant studies from China, a large country in the Asian region. This study is the first population-based retrospective cohort conducted in China, and we found that 0.74% (533) out of 72,236 pregnant women had offspring with CHD, a prevalence comparable to that reported in a national cohort in Sweden (approximately 0.80%) [19].

Several cohort studies have suggested that GDM may increase the risk of CHD in offspring [10, 20, 21], while another population-based study has reported inconsistent findings [22]. In our present study, we found that GDM and abnormal blood glucose at different time points in the OGTT were positively associated with fetal CHD risk. Moreover, there was a trend towards increased fetal CHD risk with increasing OGTT cross-group levels. These findings underscore the detrimental impact of mid-pregnancy hyperglycemia on fetal cardiac development. Notably, pregnant women with normal fasting glucose levels but abnormal OGTT 1 h or 2 h results may also be at risk of fetal congenital cardiac malformations. This group could be overlooked as a hidden risk group due to their normal fasting glucose levels and should receive appropriate attention. The reason inherent in this may be that abnormalities at different time points in the OGGT reveal glucose metabolism disorders in GDM patients. The blood glucose results of the OGTT 1 h or 2 h may reflect greater beta cell dysfunction and insulin resistance [23, 24]. Moreover, there is a requirement to consider the daily long term and hidden impairment of glucose metabolism indicated by hemoglobin A1c (HbA1c) levels [25]. Thus, only fasting blood glucose levels are inadequate for reflecting maternal glucose metabolism.

The exact mechanism by which GDM is linked to CHD in fetuses is still not fully understood and remains a mystery. Cardiogenesis begins with mesodermal cells in the second week of gestation, and the heart is among the first organs to develop during embryonic growth. During the third weeks of gestation, a linear tubular structure is formed, which becomes longer, bulging, and twisted during the fourth week of gestation. The final maturation of the heart takes place during the seventh to eighth week of gestation. GDM is typically diagnosed after 24 weeks of pregnancy, which contradicts the biological feasibility of the observed birth defects. However, women who develop GDM during pregnancy may have preexisting metabolic dysfunctions such as islet beta-cell defects and increased insulin resistance, which may cause mild hyperglycemic symptoms in early pregnancy [26, 27]. Hyperglycemia-induced cardiac abnormalities in offspring may involve multiple pathways. Oxidative stress is a crucial process in diabetes pathogenesis, and hyperglycemia can trigger the production of reactive oxygen species (ROS). Even though oxidative stress does not directly cause genotoxicity, it can influence gene expression throughout the embryonic development stages, indirectly contributing to cardiac abnormalities in offspring [28]. A study conducted on diabetic pregnant rats revealed that higher blood glucose levels during embryonic heart development resulted in increased oxidative stress and endoplasmic reticulum stress in cardiac cells, which led to excessive apoptosis of ventricular muscle and endocardial cushion cells during the embryonic period, ultimately leading to the development of CHD such as ventricular septal defects [29]. A cellular assay conducted in vitro also suggests that FoxO3a, a transcriptional regulator vital to oxidative stress, induces apoptosis in a high glucose state [30]. In addition, although glucose itself is not a mutagen, it can affect insulin sensitivity and the expression of insulin-like growth factor (IGF). Also, inhibition of IGF signaling in the mother during early pregnancy may lead to adverse pregnancy outcomes, including CHD [31]. Thus, glucose may affect IGF and play a role in CHD development. Hyperglycemia can also affect critical pathways of cardiac development by modifying the expression profile of miRNAs, leading to CHD. A study comparing miRNA expression profiles in the embryonic hearts of diabetic and non-diabetic mice found up to 167 differentially expressed miRNAs in abundance [32]. The target genes of many miRNAs can express transcription factors and are associated with cardiac dysplasia and apoptosis, such as Nkx2.5, Zeb2, Mef2c, and Ets1 [32–34]. It should be emphasised, of course, that by the time the OGTT is performed, the fetal heart formation has already developed and the results of the OGTT may not be a direct risk factor for the fetal heart defects. However, the diagnosis of maternal GDM at the time of the OGTT also reflects, to a certain extent, the metabolism of blood glucose in early pregnancy, as it is possible that GDM has already been present, but has not been detected. On the other hand, the results of the OGTT are also useful as an indicator for screening high-risk fetuses.

AVSDs are a type of congenital heart defect characterized by defects in the septum at or near the level of the AV valves, including primum atrial septal defects, inflow ventricular septal defects, and complete common AV canal spanning the atria and ventricles [35]. The severity of AVSDs can vary from partial to transitional to complete, as well as AVSDs associated with ectopic syndrome and tetralogy of Fallot, depending on the morphology and development of the atrioventricular valves [36]. The incidence of AVSD in the general population is reported to be 0.24 to 0.31 per 1000 live births [37]. The current study found a significant association between high blood glucose levels at different time points in OGTT during mid-pregnancy and an increased risk of AVSD in the offspring. This is consistent with the findings of a meta-analysis published in 2019 that reported a 4.74 times higher risk of fetal AVSD associated with maternal diabetes [18]. An animal study suggested that the developmental period of the endocardial cushion is an important time for the formation of AVSDs, and that endoplasmic reticulum stress induced by maternal hyperglycemia may play a role in this process [38].

Subgroup analysis revealed that the association between mid-pregnancy hyperglycemia and CHD risk in the offspring varied by factors such as maternal age and menstrual status. Non-advanced maternal age was found to be a high-risk group for hyperglycemia-induced CHD. However, older mothers may be more likely to receive advice from their doctors during pregnancy check-ups and therefore pay more attention to their diet and lifestyle during pregnancy, potentially reducing their risk of adverse pregnancy outcomes. Assisted reproduction and gestational hypertension may also contribute to differences in subgroup analysis due to sample size.

Several limitations of this study should be noted. The study only examined blood glucose changes from 24–28 weeks of gestation and did not record whether pregnant women took steps to control their blood glucose. Additionally, potential confounding factors such as smoking, alcohol consumption, and caffeine use were not investigated. However, according to Chinese social customs, alcohol and cigarette consumption are rare among pregnant women, who generally prefer tea. Besides, as mentioned above, HbA1c is important to evaluate the meaning of the OGTT result, but HbA1c levels were not available in this study. Finally, this was a single-center cohort study, and the data only included information from the Maternal and Child Health Hospital in Fujian Province, although this facility serves almost half of the pregnant women in the city and can be considered somewhat representative of the population.

## Conclusions

GDM, abnormal glucose regulation at different time points in OGTT associated with a higher risk of congenital heart malformations in the fetus. Furthermore, the incidence of CHD in the offspring tends to increase with increasing levels of glycemic cross-grouping. The findings also remind primary care hospitals that screening and management of GDM are not sufficiently dependent on checking fasting blood glucose alone. The offspring of pregnant women with GDM diagnosed by OGTT should be managed as high-risk fetuses for congenital heart defects.

## Data Availability

The datasets used and analyzed during the current study are available from the corresponding author on reasonable request.

## References

[1] Ferrara A (2007). Increasing prevalence of gestational diabetes mellitus: a public health perspective. Diabetes Care.

[2] Wendland EM, Torloni MR, Falavigna M, Trujillo J, Dode MA, Campos MA, Duncan BB, Schmidt MI (2012). Gestational diabetes and pregnancy outcomes–a systematic review of the World Health Organization (WHO) and the International Association of Diabetes in Pregnancy Study Groups (IADPSG) diagnostic criteria. BMC Pregnancy Childbirth.

[3] American Diabetes A (2019). 2. Classification and Diagnosis of Diabetes: Standards of Medical Care in Diabetes-2019.. Diabetes Care.

[4] Sacks DA, Hadden DR, Maresh M, Deerochanawong C, Dyer AR, Metzger BE, Lowe LP, Coustan DR, Hod M, Oats JJN (2012). Frequency of Gestational Diabetes Mellitus at Collaborating Centers Based on IADPSG Consensus Panel-Recommended Criteria. Diabetes Care.

[5] Lee KW, Ching SM, Ramachandran V, Yee A, Hoo FK, Chia YC, Wan Sulaiman WA, Suppiah S, Mohamed MH, Veettil SK (2018). Prevalence and risk factors of gestational diabetes mellitus in Asia: a systematic review and meta-analysis. BMC Pregnancy Childbirth.

[6] Darbandi M, Rezaeian S, Dianatinasab M, Yaghoobi H, Soltani M, Etemad K, Valadbeigi T, Taherpour N, Hajipour M, Saeidi R (2021). Prevalence of gestational diabetes and its association with stillbirth, preterm birth, macrosomia, abortion and cesarean delivery: a national prevalence study of 11 provinces in Iran. J Prev Med Hyg.

[7] Ornoy A, Becker M, Weinstein-Fudim L, Ergaz Z (2021). Diabetes during Pregnancy: A Maternal Disease Complicating the Course of Pregnancy with Long-Term Deleterious Effects on the Offspring. A Clinical Review. Int J Mol Sci.

[8] Zawiejska A, Wroblewska-Seniuk K, Gutaj P, Mantaj U, Gomulska A, Kippen J, Wender-Ozegowska E (2020). Early Screening for Gestational Diabetes Using IADPSG Criteria May Be a Useful Predictor for Congenital Anomalies: Preliminary Data from a High-Risk Population. J Clin Med.

[9] Papazoglou AS, Moysidis DV, Panagopoulos P, Kaklamanos EG, Tsagkaris C, Vouloagkas I, Karagiannidis E, Tagarakis GI, Papamitsou T, Papanikolaou IG (2022). Maternal diabetes mellitus and its impact on the risk of delivering a child with congenital heart disease: a systematic review and meta-analysis. J Matern Fetal Neonatal Med.

[10] Billionnet C, Mitanchez D, Weill A, Nizard J, Alla F, Hartemann A, Jacqueminet S (2017). Gestational diabetes and adverse perinatal outcomes from 716,152 births in France in 2012. Diabetologia.

[11] Brite J, Laughon SK, Troendle J, Mills J (2014). Maternal overweight and obesity and risk of congenital heart defects in offspring. Int J Obes (Lond).

[12] Liu X, Liu G, Wang P, Huang Y, Liu E, Li D, Ren S, Pan L, Li N, Yang X (2015). Prevalence of congenital heart disease and its related risk indicators among 90,796 Chinese infants aged less than 6 months in Tianjin. Int J Epidemiol.

[13] Gabbay-Benziv R, Reece EA, Wang F, Yang P (2015). Birth defects in pregestational diabetes: Defect range, glycemic threshold and pathogenesis. World J Diabetes.

[14] Passarella G, Trifiro G, Gasparetto M, Moreolo GS, Milanesi O (2013). Disorders in glucidic metabolism and congenital heart diseases: detection and prevention. Pediatr Cardiol.

[15] Priest JR, Yang W, Reaven G, Knowles JW, Shaw GM (2015). Maternal Midpregnancy Glucose Levels and Risk of Congenital Heart Disease in Offspring. JAMA Pediatr.

[16] Shaw GM, Quach T, Nelson V, Carmichael SL, Schaffer DM, Selvin S, Yang W (2003). Neural tube defects associated with maternal periconceptional dietary intake of simple sugars and glycemic index. Am J Clin Nutr.

[17] Yazdy MM, Liu S, Mitchell AA, Werler MM (2010). Maternal dietary glycemic intake and the risk of neural tube defects. Am J Epidemiol.

[18] Chen L, Yang T, Chen L, Wang L, Wang T, Zhao L, Ye Z, Zhang S, Luo L, Zheng Z (2019). Risk of congenital heart defects in offspring exposed to maternal diabetes mellitus: an updated systematic review and meta-analysis. Arch Gynecol Obstet.

[19] Oyen N, Diaz LJ, Leirgul E, Boyd HA, Priest J, Mathiesen ER, Quertermous T, Wohlfahrt J, Melbye M (2016). Prepregnancy Diabetes and Offspring Risk of Congenital Heart Disease: A Nationwide Cohort Study. Circulation.

[20] Hoang TT, Marengo LK, Mitchell LE, Canfield MA, Agopian AJ (2017). Original Findings and Updated Meta-Analysis for the Association Between Maternal Diabetes and Risk for Congenital Heart Disease Phenotypes. Am J Epidemiol.

[21] Leirgul E, Brodwall K, Greve G, Vollset SE, Holmstrom H, Tell GS, Oyen N (2016). Maternal Diabetes, Birth Weight, and Neonatal Risk of Congenital Heart Defects in Norway, 1994–2009. Obstet Gynecol.

[22] Peticca P, Keely EJ, Walker MC, Yang Q, Bottomley J (2009). Pregnancy outcomes in diabetes subtypes: how do they compare? A province-based study of Ontario, 2005–2006. J Obstet Gynaecol Can.

[23] Di Cianni G, Seghieri G, Lencioni C, Cuccuru I, Anichini R, De Bellis A, Ghio A, Tesi F, Volpe L, Del Prato S (2007). Normal glucose tolerance and gestational diabetes mellitus: what is in between?. Diabetes Care.

[24] Retnakaran R, Qi Y, Sermer M, Connelly PW, Zinman B, Hanley AJ (2008). Isolated hyperglycemia at 1 hour on oral glucose tolerance test in pregnancy resembles gestational diabetes mellitus in predicting postpartum metabolic dysfunction. Diabetes Care.

[25] Herman WH, Fajans SS (2010). Hemoglobin A1c for the diagnosis of diabetes: practical considerations. Pol Arch Med Wewn.

[26] Buchanan TA, Xiang AH, Page KA (2012). Gestational diabetes mellitus: risks and management during and after pregnancy. Nat Rev Endocrinol.

[27] McIntyre HD, Catalano P, Zhang C, Desoye G, Mathiesen ER, Damm P (2019). Gestational diabetes mellitus. Nat Rev Dis Primers.

[28] Clapes S, Fernandez T, Suarez G (2013). Oxidative stress and birth defects in infants of women with pregestational diabetes. MEDICC Rev.

[29] Wu Y, Reece EA, Zhong J, Dong D, Shen WB, Harman CR, Yang P (2016). Type 2 diabetes mellitus induces congenital heart defects in murine embryos by increasing oxidative stress, endoplasmic reticulum stress, and apoptosis. Am J Obstet Gynecol.

[30] Yang P, Yang WW, Chen X, Kaushal S, Dong D, Shen WB (2017). Maternal diabetes and high glucose in vitro trigger Sca1(+) cardiac progenitor cell apoptosis through FoxO3a. Biochem Biophys Res Commun.

[31] Bowman CJ, Streck RD, Chapin RE (2010). Maternal-placental insulin-like growth factor (IGF) signaling and its importance to normal embryo-fetal development. Birth Defects Res B Dev Reprod Toxicol.

[32] Dong D, Zhang Y, Reece EA, Wang L, Harman CR, Yang P (2016). microRNA expression profiling and functional annotation analysis of their targets modulated by oxidative stress during embryonic heart development in diabetic mice. Reprod Toxicol.

[33] Wang J, Wang F, Gui YH (2017). Research advances in the mechanism of congenital heart disease induced by pregestational diabetes mellitus. Zhongguo Dang Dai Er Ke Za Zhi.

[34] Zhao M, Diao J, Huang P, Li J, Li Y, Yang Y, Luo L, Zhang S, Chen L, Wang T (2020). Association of Maternal Diabetes Mellitus and Polymorphisms of the NKX2.5 Gene in Children with Congenital Heart Disease: A Single Centre-Based Case-Control Study. J Diabetes Res.

[35] Burnicka-Turek O, Steimle JD, Huang W, Felker L, Kamp A, Kweon J, Peterson M, Reeves RH, Maslen CL, Gruber PJ (2016). Cilia gene mutations cause atrioventricular septal defects by multiple mechanisms. Hum Mol Genet.

[36] Jacobs JP, Burke RP, Quintessenza JA, Mavroudis C (2000). Congenital Heart Surgery Nomenclature and Database Project: atrioventricular canal defect. Ann Thorac Surg.

[37] Craig B (2006). Atrioventricular septal defect: from fetus to adult. Heart.

[38] Zhao Z (2012). Endoplasmic reticulum stress in maternal diabetes-induced cardiac malformations during critical cardiogenesis period. Birth Defects Res B Dev Reprod Toxicol.

